# Photoelectric Properties of Si Doping Superlattice Structure on 6H-SiC(0001)

**DOI:** 10.3390/ma10060583

**Published:** 2017-05-25

**Authors:** Lianbi Li, Yuan Zang, Jichao Hu, Shenghuang Lin, Zhiming Chen

**Affiliations:** 1 School of Science, Xi’an Polytechnic University, Xi’an 710048, China; 2 Department of Electronic Engineering, Xi’an University of Technology, Xi’an 710048, China; zangyuan82@163.com (Y.Z.); hujichao0613@163.com (J.H.); chenzm@xaut.edu.cn (Z.C.); 3 Department of Applied Physics, The Hong Kong Polytechnic University, Hong Kong, China; shenghuang.lin@polyu.edu.hk

**Keywords:** Si/6H-SiC heterostructure, doping superlattice, photoelectric properties, chemical vapor deposition, transmission electron microscopy

## Abstract

The energy-band structure and visible photoelectric properties of a p/n-Si doping superlattice structure (DSL) on 6H-SiC were simulated by Silvaco-TCAD. The,n the Si-DSL structures with 40 nm-p-Si/50 nm-n-Si multilayers were successfully prepared on 6H-SiC(0001) Si-face by chemical vapor deposition. TEM characterizations of the p/n-Si DSL confirmed the epitaxial growth of the Si films with preferred orientation and the misfit dislocations with a Burgers vector of 1/3 <21-1> at the p-Si/n-Si interface. The device had an obvious rectifying behavior, and the turn-on voltage was about 1.2 V. Under the visible illumination of 0.6 W/cm^2^, the device demonstrated a significant photoelectric response with a photocurrent density of 2.1 mA/cm^2^. Visible light operation of the Si-DSL/6H-SiC heterostructure was realized for the first time.

## 1. Introduction

SiC is a desirable material for power devices due to its superior physical properties such as a wide bandgap, high thermal conductivity, and high critical electric field, etc. [1,2,3,4,5,6,7]. In order to realize the visible light operation of SiC photoelectric devices applied in high temperature and high power regions, Si films were grown on SiC for visible light absorption [8,9]. The SiC-based Si/SiC heterojunction is comparatively less studied [10,11,12,13]. Present studies mainly focus on using an Si inter-layer to reduce the interface states density of the SiC surface oxide layer in SiC MOSFET [12,13], and seldom focus on the aspects of light operation of SiC photoelectric devices. In our previous work, visible-light-operated Si/SiC photodiodes with PIN and Schottky structures were prepared for the first time [8,9]. However, because of the large lattice mismatch between Si and 6H-SiC (~19.6%), there was still a high density of structural defects such as edge misfit dislocations at the hetero-interface [14], stacking faults, and twins in Si films [15], which led to a small carrier lifetime and poor device properties [8,9]. Interestingly, the doping superlattice (DSL) is a structure composed of periodic p/n doped layers, in which the space-charge potential of ionized impurities modulates the bandgaps of the materials, and hence separates the electrons and holes spatially and further enhances the carrier lifetime and the photoelectric properties of the device [16,17,18,19]. Employing DSL structures based on amorphous hydrogenated silicon (a-Si:H) [16,17], an increase in the photoconductivity up to tenfold can be observed when compared to that of the unstructured a-Si:H, which paves a possible way for the fabrication of DSL structures on SiC substrates to improve the photoconductivity of SiC-based photodetectors. 

In this paper, p/n-Si DSL structures on 6H-SiC substrates were accordingly adopted to promote the photoelectric properties of the Si/SiC heterojunctions. The energy-band structure and visible photoelectric properties of the p/n-Si DSL structures on 6H-SiC were simulated by Silvaco-TCAD. Then, the visible photodetector with the p/n-Si DSL structure was prepared on 6H-SiC(0001) Si-face by low-pressure chemical vapor deposition (LPCVD). Transmission electron microscopy (TEM) and selected area electron diffraction (SAED) were employed to investigate the interface structure of the Si-DSL/SiC heterostructure, and its photoelectric properties were investigated.

## 2. Results and Discussion

### 2.1. Device Structure and the Simulation Results

**Figure 1:**
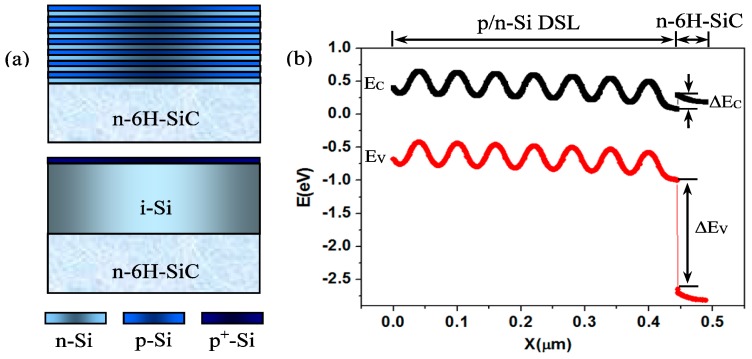
Schematic diagram of p/n-Si DSL and PIN-Si structures on 6H-SiC (**a**); energy-band diagram of p/n-Si DSL structure with seven p/n-Si junctions on 6H-SiC in a thermal equilibrium state (**b**). ΔE_C_ = 0.21 eV, ΔE_V_ = 1.65 eV.

Figure 1a shows the schematic diagram of p/n-Si DSL and PIN-Si structures on 6H-SiC. Because of the wide bandgap 6H-SiC is used as the penetration window of the visible lights in photoelectric characterization. The visible light passes through 6H-SiC freely and is absorbed in the p/n-Si DSL structures. The photoelectric properties of the p/n-Si DSL structure on 6H-SiC are simulated by Silvaco-TCAD. In simulation the electron affinity and bandgap of 6H-SiC are set as 3.85 eV and 3.0 eV [20], respectively. The impurity concentrations of the n-Si and p-Si layers are set as 10^15^ cm^−3^ and 10^16^ cm^−3^. The impurity concentration of the n-type 6H-SiC with a thickness of 300 µm is 10^17^ cm^−3^, which is consistent with the experimental parameters. Figure 1b shows the energy-band diagram of p/n-Si DSL structures on 6H-SiC in a thermal equilibrium state. It is shown that there are energy offsets of the n-type isotype Si/6H-SiC heterostructure in the conduction band ΔE_C_ and valance band ΔE_V_, which are about 0.21 eV and 1.65 eV, respectively. The electron barrier of the Si/6H-SiC heterostructure is much lower than the hole barrier, and thus the electrons dominate the current carriers.

**Figure 2:**
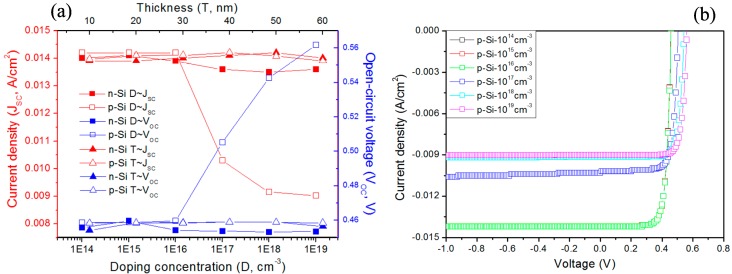
Simulated J_SC_ and V_OC_ of the p/n-Si DSL structure on 6H-SiC with different impurity concentrations and thicknesses under a visible illumination of 0.1 W/cm^2^ (**a**); J-V curves of the Si-DSL/6H-SiC heterostructure with different impurity concentrations of the p-Si layer (**b**).

Figure 2a shows the simulated photoelectric properties of the Si-DSL/6H-SiC heterostructure with different impurity concentrations and thicknesses. With the decrease of the p/n-Si impurity concentration the photocurrent density J_SC_ increases. This is due to the widen space-charge region, which leads to the low recombination of the photogenerated carriers. In particular, the impurity concentration of the p-Si layer has an important influence on the photoelectric properties, as shown in Figure 2b. As the impurity concentration of the p-layer decreases, the J_SC_ increases rapidly, and the turn-on voltage V_OC_ decreases correspondingly. Compared with the impurity concentration, the thickness has a relatively weak influence on the photoelectric properties. It is shown that the photocurrent density increases slightly and then declines as the thickness increases. Under a visible illumination of 0.1 W/cm^2^, I_SC_ reaches the largest value as the impurity concentrations are 10^15^ cm^−3^ and 10^16^ cm^−3^, while the thickness of the n-layer and p-layer are 50 nm and 40 nm, respectively.

**Figure 3:**
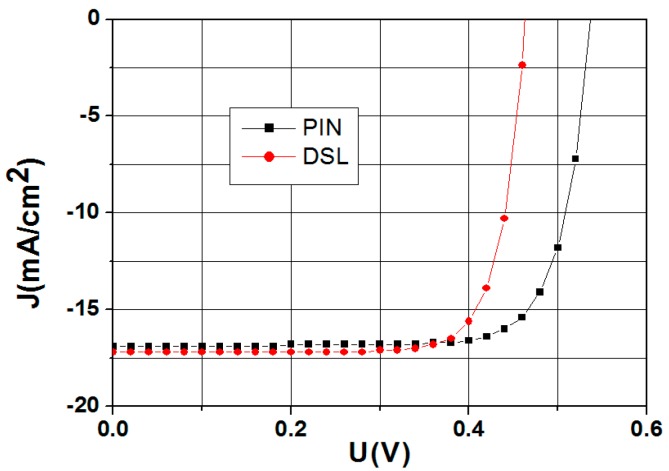
Simulated J-V curves of Si-DSL/6H-SiC heterostructure and PIN Si/6H-SiC heterostructure under a visible illumination of 0.1 W/cm^2^.

The photoelectric properties of the optimized Si-DSL/6H-SiC heterostructure are compared with the PIN Si/6H-SiC heterostructure. When the impurity concentrations of the n-layer and p-layer are 10^15^ cm^−3^ and 10^16^ cm^−3^, the I_SC_ of the DSL structure is 3.0% higher than that of the PIN Si/6H-SiC heterostructure under a visible illumination of 0.1 W/cm^2^, as shown in Figure 3. It is demonstrated that the Si-DSL/6H-SiC heterostructure has more advantages than the PIN Si/6H-SiC structure in the photoelectric response, especially in the case of small carrier lifetime. Furthermore, the PIN Si/6H-SiC heterostructure has the higher V_OC_. This is due to the high impurity concentration of the p-Si layer, which results in a higher electron barrier.

### 2.2. Experiments and Results Discussion

The p/n-Si DSL structures are grown on n-type 6H-SiC(0001) Si-face by LPCVD. An n-type doped (impurity concentration of ~10^17^ cm^−3^) 6H-SiC(0001) wafer with a thickness of 300 μm was purchased from II-VI Inc. (Saxonburg, PA, USA). Silane (SiH_4_), diborane (B_2_H_6_), and hydrogen (H_2_) are used as a silicon source, a p-type doping source, and a carrier, respectively. After the standard Radio Corporation of America (RCA) cleaning processes, 6H-SiC substrates were treated in high-purity H_2_ at 1050 °C for 10 min. Then, the p/n-Si DSL structures with seven p/n-Si junctions were prepared at 900 °C. To prepare the ohmic contacts, Al electrodes on Si and Ni electrodes on 6H-SiC were prepared by magnetron sputtering followed by annealing at 900 °C and 1050 °C, respectively.

**Figure 4:**
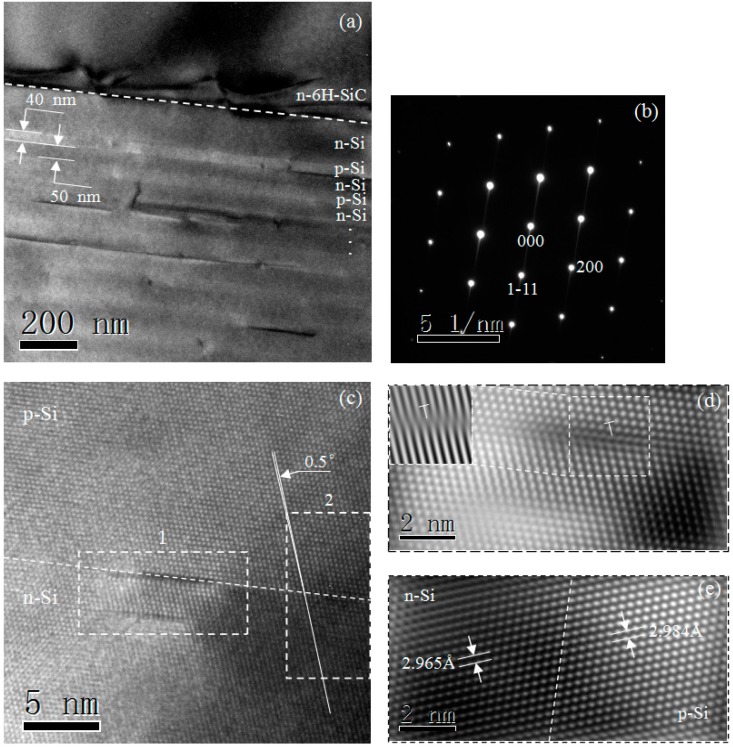
TEM and selected area electron diffraction (SAED) images of the Si-DSL structures with seven p/n-Si junctions on 6H-SiC. (**a**) Cross-sectional low magnification TEM image; (**b**) SAED patterns of the Si films; (**c**) HRTEM image of the p-Si/n-Si interface; and the processed HRTEM images of region 1 (**d**) and region 2 (**e**) by using the Fourier filtering technique.

The low magnification cross-sectional TEM bright-field image of the Si-DSL/6H-SiC heterostructure is shown in Figure 4a. The p/n-Si DSL structures with obvious contrast differences show 40 nm-p-Si/50 nm-n-Si multilayers on 6H-SiC(0001). The p-Si/n-Si interface is abrupt in structure, and the p-Si layers exhibit brighter contrast. The SAED patterns of the p/n-Si DSL corresponding to the Si[011] zone axes confirm the epitaxial growth of the Si films with [1,2,3,4,5,6,7,8,9,10,11] preferred orientation, as shown in Figure 4b. Figure 4c shows a high-resolution TEM image of the p-Si/n-Si interface. There are some structural defects at the interface. The Si[111] orientation was tilted by 0.5° at the interface. Fourier filtering technique (FFT) is applied to reveal the periodic atomic arrangement of the p-Si/n-Si interface. Figure 4d shows the enlarged FFT image of region 1. Relatively small amounts of misfit dislocations (MD) are observed at the p-Si/n-Si interface, which can be easily identified by extra lattice fringes in the p-Si layer. The MDs are of the pure edge type with a Burgers vector of 1/3 <21-1> parallel to the interface, and are labelled in Figure 4d. Moreover, the crystal plane spacing of the Si film has relatively obvious change at the p-Si/n-Si interface, as shown in Figure 4e. The crystal plane spacing of p-Si is 0.64% lower than the n-Si layer. This demonstrates that the occurrence of the interfacial MDs cannot accommodate most of lattice mismatch strain and cause the lattice to change at the p/n-Si DSL interface.

**Figure 5:**
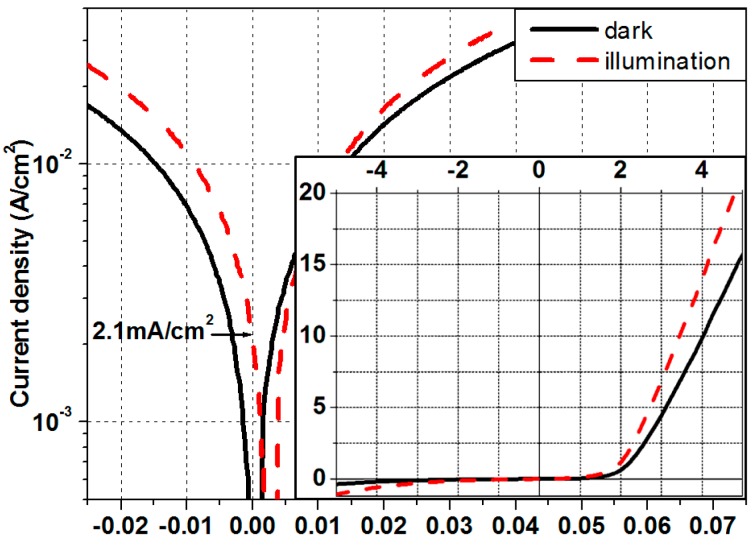
Photoelectric properties of the p/n-Si DSL structures with seven p/n-Si junctions on 6H-SiC.

Figure 5 presents the photoelectric properties of the Si-DSL/6H-SiC heterostructure. The heterostructure has obvious rectifying characteristics with a rectifying ratio up to 40 at ± 5 V, and the turn-on voltage is about 1.2 V, as shown in the inset. Under a visible illumination of 0.6 W/cm^2^, the heterostructure demonstrates apparent photoelectric behavior with a J_SC_ of ~2.1 mA/cm^2^. Visible light operation of the Si-DSL/6H-SiC heterostructure is realized. However, compared with the simulated results, the device demonstrates a smaller J_SC_ and a large reverse leakage current. When the reverse voltage is 5 V, the leakage current density is as large as 0.36 A/cm^2^. This is due to the structural defects such as the MDs at the p-Si/n-Si interface, which result in large recombination current and deteriorating device properties.

## 3. Conclusions

In this paper, the energy-band structure and visible photoelectric properties of an Si-DSL doping superlattice structure on 6H-SiC were simulated by Silvaco-TCAD. The Si-DSL structures with 40 nm-p-Si/50 nm-n-Si multilayers were successfully prepared on 6H-SiC(0001) Si-face by LPCVD. The energy offsets of the n-Si/n-6H-SiC heterojunction in the conduction band and valance band were calculated to be 0.21 eV and 1.65 eV, respectively. The electrons dominate the current carriers because of the low electron barrier. TEM characterizations of the p/n-Si DSL confirmed the epitaxial growth of the Si films with [1,2,3,4,5,6,7,8,9,10,11] preferred orientation and the misfit dislocations with a Burgers vector of 1/3 <21-1>at the p-Si/n-Si interface. Under a visible illumination of 0.6 W/cm^2^, the heterostructure demonstrates apparent rectifying behavior and significant photoelectric response with a photocurrent density of 2.1 mA/cm^2^. Visible light operation of the Si-DSL/6H-SiC heterostructure was successfully realized.
